# Rhizosphere community selection reveals bacteria associated with reduced root disease

**DOI:** 10.1186/s40168-020-00997-5

**Published:** 2021-04-09

**Authors:** Chuntao Yin, Juan M. Casa Vargas, Daniel C. Schlatter, Christina H. Hagerty, Scot H. Hulbert, Timothy C. Paulitz

**Affiliations:** 1 Department of Plant Pathology, Washington State University, Pullman, WA 99164-6430 USA; 2 USDA-ARS, Wheat Health, Genetics and Quality Research Unit, Washington State University, Pullman, WA 99164-6430 USA; 3 Columbia Basin Agricultural Research Center, Oregon State University, Adams, OR 97810 USA

**Keywords:** Microbial community, *Rhizoctonia solani*, Disease suppression, Antagonism, Beneficial bacteria

## Abstract

**Background:**

Microbes benefit plants by increasing nutrient availability, producing plant growth hormones, and protecting against pathogens. However, it is largely unknown how plants change root microbial communities.

**Results:**

In this study, we used a multi-cycle selection system and infection by the soilborne fungal pathogen *Rhizoctonia solani* AG8 (hereafter AG8) to examine how plants impact the rhizosphere bacterial community and recruit beneficial microorganisms to suppress soilborne fungal pathogens and promote plant growth. Successive plantings dramatically enhanced disease suppression on susceptible wheat cultivars to AG8 in the greenhouse. Accordingly, analysis of the rhizosphere soil microbial community using deep sequencing of 16S rRNA genes revealed distinct bacterial community profiles assembled over successive wheat plantings. Moreover, the cluster of bacterial communities formed from the AG8-infected rhizosphere was distinct from those without AG8 infection. Interestingly, the bacterial communities from the rhizosphere with the lowest wheat root disease gradually separated from those with the worst wheat root disease over planting cycles. Successive monocultures and application of AG8 increased the abundance of some bacterial genera which have potential antagonistic activities, such as *Chitinophaga*, *Pseudomonas*, *Chryseobacterium*, and *Flavobacterium*, and a group of plant growth-promoting (PGP) and nitrogen-fixing microbes, including *Pedobacter*, *Variovorax*, and *Rhizobium*. Furthermore, 47 bacteria isolates belong to 35 species were isolated. Among them, eleven and five exhibited antagonistic activities to AG8 and *Rhizoctonia oryzae in vitro*, respectively. Notably, *Janthinobacterium* displayed broad antagonism against the soilborne pathogens *Pythium ultimum*, AG8, and *R. oryzae in vitro*, and disease suppressive activity to AG8 in soil.

**Conclusions:**

Our results demonstrated that successive wheat plantings and pathogen infection can shape the rhizosphere microbial communities and specifically accumulate a group of beneficial microbes. Our findings suggest that soil community selection may offer the potential for addressing agronomic concerns associated with plant diseases and crop productivity.

Video Abstract

**Supplementary Information:**

The online version contains supplementary material available at 10.1186/s40168-020-00997-5.

## Background

It is widely accepted that the rhizosphere microbial communities are tightly associated with plant roots [1, 2]. The microbiota can be harmful or beneficial to the host plant. Soilborne pathogens reduce plant growth, cause yield loss, and threaten agricultural production. However, nonpathogenic microbes, such as beneficial and mutualistic microbes, can promote plant growth by increasing nutrient availability, producing plant hormones, enhancing tolerance to abiotic stresses, and adapting to environmental variations [2–7]. Moreover, beneficial microbes can protect plants against pathogens through antagonism, competition, or by interfering with the host immunity to establish a mutualistic association with the host [8–17]. Recent work has suggested that members of the plant microbiota can enhance host immune functions [18]. Therefore, understanding how root microbiota influence plant performance is of great agronomic interest.

In return, microbial community establishment in the rhizosphere is not random but rather driven by host plant selection [1, 14, 15]. Plants have the capacity to change soil microbiota by secreting bioactive molecules into the rhizosphere. Different plant species can influence microbiome composition and structure, and some species appear to have a much stronger association with specific microbes than others [19, 20]. The effects of plant genotype on the rhizobiome have been reported in *Arabidopsis*, cucumber, bean, *Brachypodium*, maize, barley, and wheat [21–26], though the intra-specific genetic contribution to microbiome assembly is relatively low. Decades of research have shown that plant genotype/species and developmental stages impact the qualitative and quantitative composition of plant root exudates [21, 27–30]. The plant may not be the only determinant in root exudates. Various abiotic and biotic factors, such as soil type and pathogens, in the surrounding environment also influence root exudates [28, 31]. Root exudates are released into the rhizosphere where they are crucial in attracting and selecting microorganisms, thus altering the composition and structure of rhizosphere microbial communities in a plant-specific manner [32]. Plants specifically attract beneficial microbes through plant root-derived signals [33]. Furthermore, plant immune systems might promote the accommodation and growth of beneficial microbes and contribute to the maintenance of a stable microorganism community, thus playing an important part in regulating variations in microbiota composition [34]. Tkacz et al. [28] used a successive planting approach to investigate shifts in the microbial populations in the soil and found that *Arabidopsis*, *Medicago*, and *Brachypodium* select soil microbiomes differently. Similarly, Panke-Buisse et al. [35] revealed distinct microbiota profiles assembled by *Arabidopsis thaliana* at various flowering time treatments using a multi-generation experimental system. However, the effects of plants on the composition of rhizosphere communities are highly complex and dynamic. Our understanding of how plants shape rhizosphere microorganism assembly is still not fully understood.

Microbial pathogens can cause severe damage to plant roots resulting in significant agricultural yield loss. Intriguingly, continuous growth of a susceptible plant in the field and a disease outbreak often induce suppression of soilborne fungal pathogens by altering soil microbial community. And similar phenomena appear to occur in different soils from geographically distinct regions that suggests disease suppression is developed through similar mechanisms [16, 36, 37]. Several studies revealed that plants respond to pathogen attack by producing chemical compounds which attract a suit of beneficial microorganisms [38, 39]. Similarly, Rudrappa et al. [31] demonstrated that *Arabidopsis thaliana* selectively recruit the beneficial bacterium *Bacillus subtilis* when challenged with pathogen *Pseudomonas syringae* pv. *tomato* DC3000. Microbial network analysis found microbial taxa that were positively associated with the absence of root infection by *Rhizoctonia solani* [40]. In Dudenhöffer’s study, barley plants manipulated their rhizosphere community to recruit antifungal microbes in response to *Fusarium graminearum* attack [41]. Taken together, these discoveries indicate a tight linkage between the microbial community in planta and pathogen infection and provide the possibility that plants recruit disease-suppressive microbes in response to pathogen attack.

In this study, we conducted multi-cycle wheat plantings with or without *Rhizoctonia solani* AG8 infection to address (1) whether multi-cycle wheat plantings and pathogen infection influence the structure of rhizosphere bacterial communities; (2) whether the assembly of rhizosphere bacterial communities affect plant disease development caused by AG8; and (3) whether microbial recruitment is associated with antifungal activities. Overall, we hypothesized that plant rhizosphere succession and interaction of host and soilborne pathogens might manipulate their rhizosphere microbiome structure, and recruit/enrich beneficial or antagonistic microorganisms to suppress pathogens in the rhizosphere, eventually providing a new opportunity to suppress root disease and increase crop production.

## Methods

### Plant and growth conditions

The wheat cultivar Alpowa, highly susceptible to *Rhizoctonia solani* AG8, was used in this study. All wheat seeds were derived from the same seed source to reduce plant variation. Seeds were treated with 5% sodium hypochlorite for 3 mins for surface disinfestation and rinsed three times with sterilized ddH_2_O before germination. All plants were grown in a growth chamber in 16/8 h light/darkness at 16 °C.

### Multi-cycle wheat plantings and microbiome selection

The soil used in this study was collected from the Washington State University Dryland Research Station near Lind (47° 0′ N, 118° 34′ W), WA, USA. Winter pea (*Pisum sativum* L.) was planted in September 2016 at the study site and plowed down in the summer of 2017. The soil was a Shano silt loam as described by Sharratt and Schillinger [42]. All soil was transferred to the greenhouse and air-dried at room temperature, pooled, and sieved through a 0.5-cm mesh screen to remove plant debris and stored in a cold room (4^o^C) for further use.

The inoculum of *R. solani* AG8 was prepared with twice-autoclaved millet seeds. Briefly, AG8 was grown in potato dextrose agar medium (PDA, Sigma-Aldrich, St. Louise, MO) for one week. The inoculated fungal agar was then added into autoclaved millet seeds in a 1-L Erlenmeyer flask and kept at room temperature in the dark for 3–4 weeks. Flasks were shaken every week to ensure even colonization of the millet seeds. Colonized millet seeds were air-dried and ground. The inoculum density was enumerated by fungal colony counts before use by dilution plating on water agar.

**Fig. 1:**
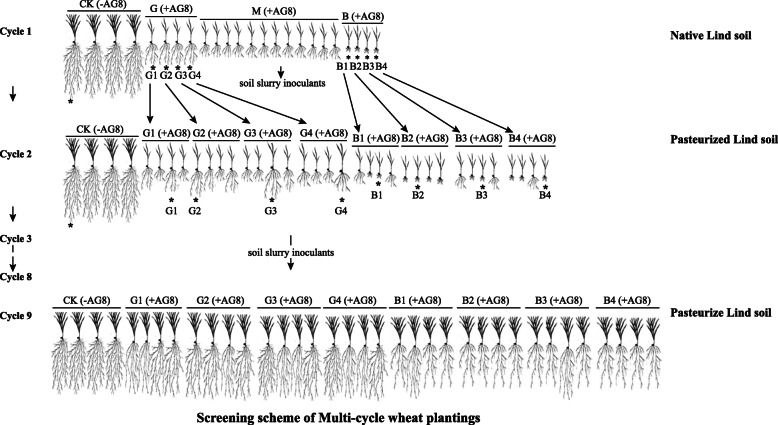
The screening scheme of multi-cycle wheat plantings in this study. The asterisk indicates the plants were chosen to prepare the soil slurry inoculants for the following cycle. CK: control plants without *Rhizoctonia solani* AG8 infection; B: plants with “bad” treatment (the worst wheat root disease); G: plants with “good” treatment (the least wheat root disease); M: plants with moderate root disease

Multi-cycle wheat plantings were conducted as described in Fig. 1. Briefly, for the first cycle (cycle 1) of plant growth, the Lind soil was amended with ground millet inoculum of AG8 to final concentration 200 propagules per gram (ppg) of soil. Twenty plastic cones (2.5 cm in diameter and 16.5 cm long) were filled with 120 g of *Rhizoctonia-*inoculated soil. Four cones filled with 120 g of soil and amended with ground autoclaved millet seeds without *Rhizoctonia* served as controls. In total, 24 cones filled with *Rhizoctonia*-inoculated soil or without *Rhizoctonia*-inoculated control soil were included. Three pre-germinated wheat seeds (cultivar Alpowa) were sown in each cone. Cones were arranged in a randomized complete block design in plastic racks and incubated in a growth chamber in 16/8 h light/darkness at 16 ^°^C. For the first 4 days, sample cones were covered with plastic. After the plastic was removed, each cone received 10 ml of water twice a week and diluted (1:3 [vol/vol]) Hoagland’s solution once a week. After 4 weeks, the wheat seedlings were removed from the cones and roots were shaken to remove adhering soil. Roots were cut and placed in a 250-ml sterile flask. Soil slurries were prepared with 1 g fresh roots and 6 ml of sterile double-distilled water at a ratio of 1:6, then vortexed for 30 s until the roots were visibly clear from adhering soil. The plants were evaluated for *Rhizoctonia* disease root rot severity on a scale of 0 to 8 as described previously [43]. Two milliliters of the soil slurries were stored at − 20 °C for DNA extraction and designated as cycle 1 samples. Among them, the soil slurries from plant growth in *Rhizoctonia*-inoculated soil and without *Rhizoctonia*-inoculated control soil were designated as “R” and “CK,” respectively. Furthermore, four soil slurries from plants with the least *Rhizoctonia* disease symptoms and four with the worst disease symptoms chosen from the 20 “R” replicates were used as microbial inoculum for the following cycle and designated as “good” (G; least disease) and “bad” (B; worst disease) treatments, respectively. The soil slurries from plants with moderate disease symptoms (M) were not kept. One soil slurry was chosen from the four “CK” replicates. Starting from the second cycle (cycle 2), the Lind soil was pasteurized at 60 °C for 30 min to reduce the inference from other microorganisms and air-dried at room temperature. The pasteurized soil amended with 200-ppg AG8 was divided into eight 480-g units for the treatment soil and one 480 g of pasteurized soil amended with ground autoclaved millet without *Rhizoctonia* served as control, then each unit received 48 ml of the corresponding treatment soil slurry inoculants from cycle 1, respectively. One hundred twenty grams of each unit soil was added to four individual cones, respectively. There were 36 cones in total, four “good” (G) and four “bad” (B) treatments, each of them having four sub-replicates, and four controls (CK). A subsequent cycle of wheat growth was initiated. After 4 weeks of growing, similar to cycle 1, the plants were evaluated for *Rhizoctonia* disease symptoms. Seedlings with the least *Rhizoctonia* disease symptoms were chosen from four sub-replicates of “good” treatments, and the worst *Rhizoctonia* disease symptom seedlings were chosen from four sub-replicates of “bad” treatments. One cone of seedlings was chosen from four control replicates. Then, the soil slurry inoculants were prepared from those chosen plant roots for the following cycle. Two milliliters of the rhizosphere soil slurries from each sample were stored at – 20 °C for rhizosphere soil DNA extraction and designated as cycle 2 samples. Cycle 3 was performed as cycle 2. Over nine cycles, 2 ml of the rhizosphere soil slurries were stored at − 20 °C for soil DNA extraction and designated as cycle 9 samples. In addition, the other 2 ml of the rhizosphere soil slurries from the cycles 5, 6, 7, 8, and 9 were used for bacteria isolation.

### DNA extraction and sequencing

DNA was extracted from 2 ml of the rhizosphere soil of cycle 1, 2, and 9 samples using a DNeasy PowerSoil kit (Qiagen, Carlsbad, CA) with the alternative protocol for wet soil samples and a FastPrep bead beater (MP Biomedical, Santa Ana, CA) using the “soil” program. The DNA was quantified using a Nanodrop spectrophotometer (Thermo Fisher Scientific, Waltham, MA) and sent to the University of Minnesota Genomics Center (UMGC) for amplification and sequencing. The V1-V3 hypervariable region of the 16S rRNA gene was amplified with primers MN_27F (5′-AGAGTTTGATCMTGGCTCAG-3′) and MN_534R (5′-ATTACCGCGGC TGCTGG-3′) using a dual-indexing approach. The detailed information on PCR was provided in Additional File 1. Then, the amplicons were pooled, size selected, spiked with 20% PhiX and sequenced for paired end (2 × 300 bp) on the Illumina MiSeq platform. The raw sequence data was deposited in the Small Read Archive of the National Center for Biotechnology Information under accession number PRJNA578725.

### Sequence processing

The sequence processing was conducted using USEARCH (version 11 [44];) to denoise sequences and define operational taxonomic units (OTUs). Specifically, reads were merged (maximum differences = 15, minimum percent ID = 80%) and primers were trimmed from the end of each read. To generate high-quality reads for denoising, reads were filtered with a maximum expected error rate of 1 and unique sequences were denoised using the “unoise3” algorithm. An OTU table was constructed by mapping all sequences to OTU representatives at a 97% similarity threshold. Taxonomy was assigned to OTUs using the SINTAX algorithm [45] with an 80% confidence threshold to the Ribosomal Database Project reference database (version 16) [46]. Sequences that could not be classified as bacteria and those identified as Streptophyta were removed and OTU tables were subsampled in the place of rarefied sequences for all analyses unless otherwise noted.

### Isolation and characterization of bacteria from rhizosphere soil

Bacteria were isolated from rhizosphere soil collected from cycles 5, 6, 7, 8, and 9, as described by Yin et al. [43]. Briefly, the rhizosphere soil slurries were serially diluted to 10-fold in 1.7-ml Eppendorf tubes. The resulting dilutions were plated on 1/4 tryptic soy agar (TSA) medium (Becton Dickinson [BD], Franklin Lakes, NJ). Because some bacteria grow poorly in the TSA medium, an R2A medium (ThermoFisher Scientific, Waltham, MA) was also used. Plates were incubated in the dark at room temperature and bacterial colonies in the plates were checked two days later. Representative colony types were picked from the most dilute plate and re-streaked on the plates with a new medium to obtain pure colonies. Colony PCR was performed with the primers MN_27/MN_543R as follows: an initial denaturation at 95 °C for 10 min, 30 cycles of 95 °C for 1 min, 55 °C for 1 min, and 72 °C for 1 min, with a final extension at 72 °C for 5 min. The PCR products were analyzed on 1.5% agarose gel electrophoresis and the approximate 600-bp amplicons were purified with the GeneJET PCR Purification Kit (ThermoFisher Scientific, Waltham, MA). The purified amplicons were sequenced by Elim Biopharmaceuticals, Inc (Hayward, CA). The sequences were blasted to the NCBI database and the Ribosomal Database Project reference database (version 16) for bacterial taxa classification. The sequence of individual bacterial isolates was blasted to the sequences of OTU representatives derived from 16S rRNA sequences and assigned to the corresponding OTU. Identified bacterial isolates were stored in 25% glycerol at − 80 °C for further use.

### In vitro antagonistic activities of bacteria against soilborne pathogens

The antagonistic activities of bacterial isolates against soilborne pathogens, including fungi *Rhizoctonia solani* AG8, *R. oryzae*, and oomycete *Pythium ultimum*, were tested by *in vitro* dual culture assays on 1/4 TSA or R2A medium, as described by Yin et al. [43]. For negative controls, petri dishes were inoculated only with an agar disc colonized with the tested pathogens. Paired culture plates were placed in the dark and incubated at 25 °C until the TSA or R2A medium for the controls was completely covered with pathogen mycelia. The radial growth of the pathogen was measured with a ruler. The percent inhibition of radial growth was calculated as follows: 100 × [(R1 − R2)/R1], where R1 was the radial growth of pathogens in the control and R2 was the radial growth of pathogens in the dual culture with the antagonist. The experiment was repeated three times with three replicates of each treatment. Bacterial isolates that showed antagonistic activity were selected for further assays.

### Greenhouse suppression assays

Bacteria that showed antagonistic activity in dual culture assay were tested against *R. solani* AG8 in soil in the greenhouse. The Lind soil was amended with ground millet inoculum of AG8 to a final concentration of 100 ppg of soil. Plastic cones (2.5 cm in diameter and 16.5 cm long) were filled with 120 g of *Rhizoctonia*-inoculated soil. The bacteria were scraped from 1/4 TSA plates, suspended in double-distilled water, and centrifuged for 3 min at 13,000 rpm. The pellet was resuspended in sterile ddH_2_O and adjusted to the optical density OD600 value of 1.0. Three-day pre-germinated wheat seeds were incubated in the bacterial slurries for 30 min at 25 °C, while the control seeds were treated with an equal amount of sterile water before planting. Wheat seeds were treated with the bacterial slurries or sterile ddH_2_O and soil samples amended with ground autoclaved millet without *Rhizoctonia* served as controls. Three wheat seeds (cultivar Alpowa) were sown in each cone. Cones were arranged in a randomized complete block design in plastic racks and incubated in a growth chamber 16/8 h light/darkness at 16 °C. Each cone received 10 ml of water twice a week and diluted (1:3 [vol/vol]) Hoagland’s solution once a week. After 3 weeks, the seedlings were removed from the cones, the roots were washed free of soil, and the fresh weight, length of the shoots, and fresh weight of root were measured. Each treatment had 6 replicates, and the experiment was conducted three times.

### Statistical analysis

Nonmetric multidimensional scaling (NMDS) and PERMANOVA were performed on Bray-Curtis dissimilarities to assess the significance of planting cycles and *R. solani* AG8 infection on bacterial community structure using the metaMDS and “Adonis” functions of the vegan package (version 2.4.1) in R (version 3.6.3) [47]. ANOVA on log2(1 + *x*)-transformed sequence counts of abundant (> 1% of total rarefied sequences) were used to examine significant differences in bacterial families among treatments (*p* ≤0.05) and heatmap was generated using ggplot2 package in R [48]. Bacterial richness and diversity metrics (Shannon’s [*H*] and inverse Simpson’s [1/*D*]) were estimated and compared among treatments using analysis of variance (ANOVA) followed by Tukey’s honest significant difference (HSD) post hoc tests (Tukey’s test). Differences in the relative abundances of bacterial genera in the rhizosphere soil between “good” (G) (the least wheat root disease) and “bad” (B) treatments (the worst wheat root disease) of successive planting cycles were assessed using DESeq2 analysis started with unrarefied OTU tables [49]. Briefly, unrarefied OTU tables were filtered to remove low abundance taxa (< 10 total) and kept OTUs with normalized counts of > 5 and that were present in three or more samples. Wald’s test was used to contrast “good” and “bad” treatment in cycles 2 and 9. Genera were counted by OTUs and considered differentially abundant if they had a base mean > 50, false discovery rate adjusted *p* <0.1, and estimated log2-fold change > 1. Multiple comparisons were performed using Tukey test in JMP (SAS Institute, Cary, NC) to identify differentially abundant bacterial taxa that were impacted by planting cycles and *R. solani* AG8 infection. Significance was accepted at an alpha level of ≤ 0.05.

## Results

### Soil bacterial community structure and composition

**Fig. 2:**
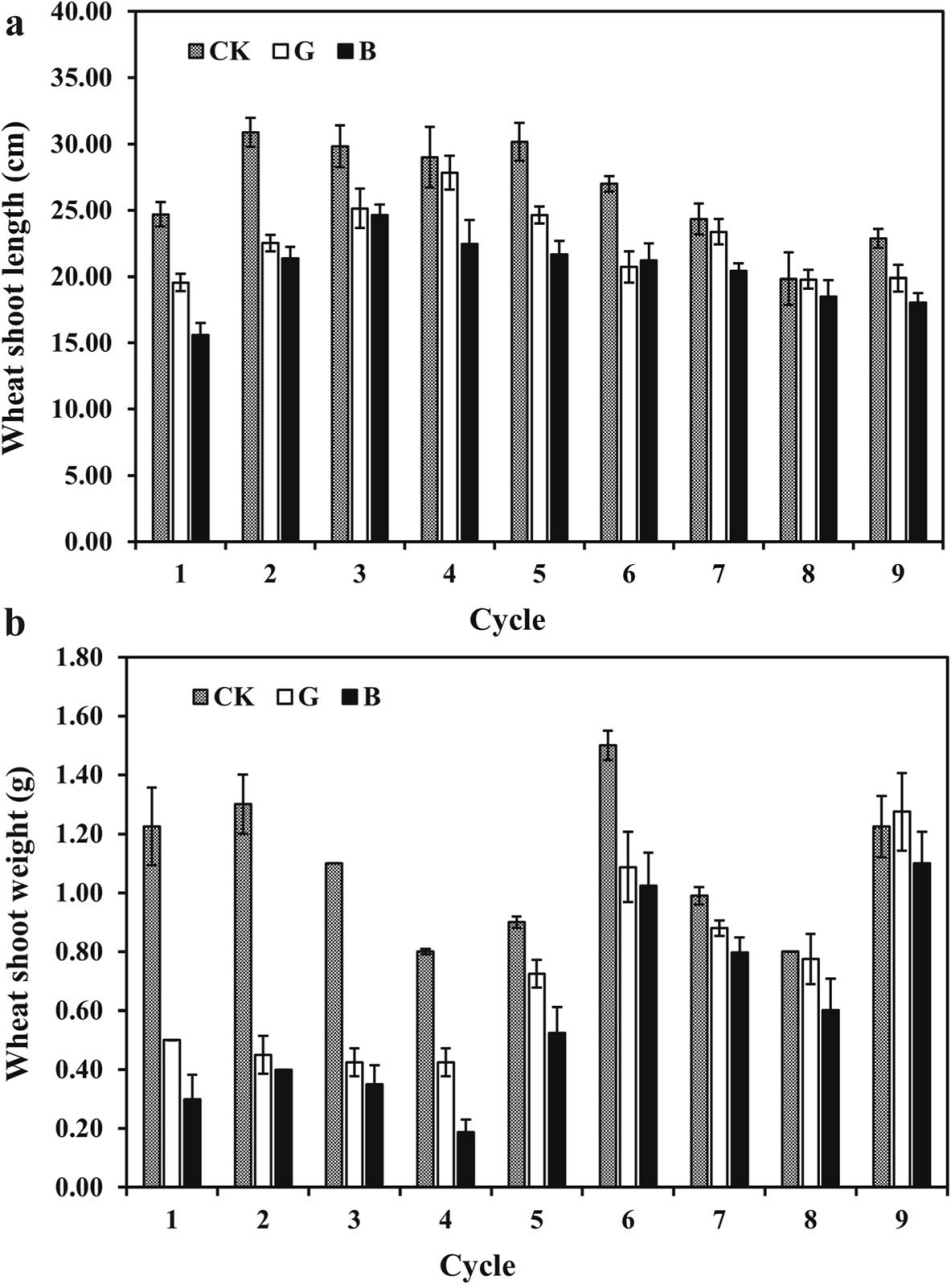
The length and weight of fresh wheat shoot over wheat growth cycles. **a** The length of fresh wheat shoot. **b** The weight of fresh wheat shoot. CK: control plants without *Rhizoctonia solani* AG8 infection; B: plants with “bad” treatment (the worst wheat root disease); G: plants with “good” treatment (the least wheat root disease)

Severe wheat shoot stunting and root damage were observed in inoculated treatments compared with wheat growth without *Rhizoctonia solani* AG8 infection in the first planting cycle (cycle 1, Fig. 2, Additional file: Fig. S1). The severe wheat root damage and shoot stunting from AG8-infected plants were still obvious in the second cycle (cycle 2), compared with the controls (Fig. 2, Additional file: Fig. S2). However, starting from the fifth cycle (cycle 5), disease suppression on wheat was developed and there was stronger suppression in “good” treatment than in the “bad” treatment, and variation of disease symptoms among sub-replicates was observed (Additional file: Fig. S3). To reduce this variation of each sub-replicate, a total of nine such cycles were conducted. In the ninth planting cycle (cycle 9), both “good” and “bad” treatments showed clear disease suppression. The difference between “good” and “bad” treatments was reduced, with no significant difference in the fresh weight and length of the shoot at cycle 9 (Fig. 2, Additional file: Fig. S4), while the measurements were more uniform among the sub-replicates.

**Fig. 3:**
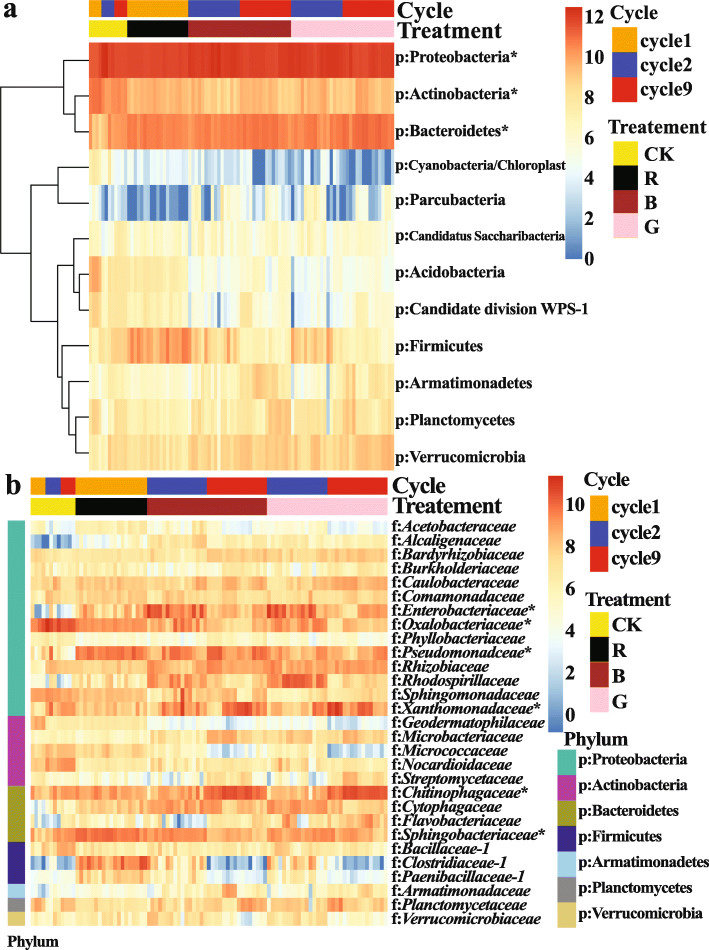
Heatmap of abundant bacterial taxa (Log2(1 + *x*)-transformed sequence counts) which were clustered based on complete-linkage hierarchical clustering of Euclidean distances. **a** Heatmap of abundant phyla. **b** Heatmap of abundant families. CK: rhizosphere soil from control plants without *R. solani* AG8 infection; R: rhizosphere soil from plants infected with *R. solani* AG8 in cycle 1; B: rhizosphere soil from plants with “bad” treatment (the worst wheat root disease); G: rhizosphere soil from plants with “good” treatment (the least wheat root disease). The asterisk indicates the most abundant phylum or families

Soil microbiota were characterized from the rhizosphere soil of the *R. solani* AG8-infested (R) and non-infested controls (CK) in cycle 1, and the “good” (G) and “bad” (B) treatments and controls (CK) in cycles 2 and 9. A total of 2,674,677 sequences were obtained and represented by 7658 OTUs. Bacterial communities were dominated by phyla Proteobacteria (50.52% ± 0.75%, mean ± SE, relative abundance among all samples), Bacteroidetes (19.87% ± 0.50%), and Actinobacteria (9.54% ± 0.47%) (Fig. 3a). The most abundant families included *Chitinophagaceae* (9.02% ± 0.54%), *Sphingobacteriaceae* (7.23% ± 0.29%), *Oxalobacteraceae* (7.21% ± 0.53%), *Xanthomonadaceae* (7.17% ± 0.56%), *Pseudomonadaceae* (7.07% ± 0.40%), and *Enterobacteriaceae* (6.33% ± 0.53%) (Fig. 3b).

### Bacterial community responses to multi-cycle plantings and *Rhizoctonia solani* AG8 infection

**Fig. 4:**
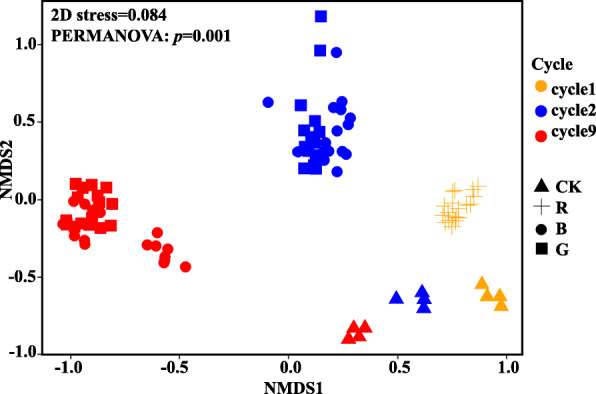
NMDS of all samples colored by planting cycles and *Rhizoctonia solani* AG8 infection (stress = 0.084). CK: rhizosphere soil from control plants without *R. solani* AG8 infection; R: rhizosphere soil from plants infected with *R. solani* AG8 in cycle 1; B: rhizosphere soil from plants with “bad” treatment (the worst wheat root disease); G: rhizosphere soil from plants with “good” treatment (the least wheat root disease)

Bacterial communities clustered clearly by planting cycles and *R. solani* AG8 infection (Fig. 4). The bacterial communities from the AG8-infected wheat rhizosphere formed clusters distinct from those without AG8 infection, and AG8 infection further enhanced bacterial community separation among planting cycles. Interestingly, the bacterial communities from “good” (G) treatment (the rhizosphere with the least wheat root disease) gradually separated from those with “bad” (B) treatment (the worst wheat root disease) over successive planting cycles although there was still overlap in cycle 9 (Fig. 4). Permutational multivariate analysis of variance (PERMANOVA) supported the effect of multi-cycle plantings and AG8 infection on bacterial communities (Table 1).

**Table 1:** PERMANOVA of impacts of plantings cycles and *R. solani* AG8 infection on bacterial communities

Factor	***F*** value	***r***^**2**^	***p*** value ^**a**^
Cycle (cycle 1, 2, and 9)	17.44	0.19	0.001 ***
Treatment (CK, R, B, and G)	18.17	0.29	0.001 ***
Cycle (cycle 1, 2, and 9) × treatment (CK, R, B, and G)	5.29	0.06	0.001 ***

*CK* rhizosphere soil from control plants without *R. solani* AG8 infection, *R* rhizosphere from plants infected with *R. solani* AG8 in the cycle 1, *B* rhizosphere from plants with “bad” treatment (the worst wheat root disease), *G* rhizosphere soil from plants with “good” treatment (the least wheat root disease). *P* values are based on 1000 permutations

^a^Significance codes: 0 ;“***” 0.001; “**” 0.01; “*” 0.05; “.” 0.1; “ ” 1

As with bacterial community structure, bacterial richness and diversity were significantly reduced with increasing planting cycle (*p* ≤ 0.05, Tukey’s test). Moreover, the bacterial richness and diversity tended to be higher in control (CK) and AG8 infection (R) in cycle 1. However, the richness and diversity did not display significant differences between the “good” (G) and “bad” (B) treated bacterial communities (Table 2).

**Table 2:** Analysis of variance of richness and diversity indices

Factors		Shannon	Simpson	Richness
Cycle	Cycle 1	6.56 ± 0.06 a	245.38 ± 27.44 a	2012.74 ± 30.35 a
	Cycle 2	5.61 ± 0.06 b	99.11 ± 7.30 b	1202.42 ± 35.63 b
	Cycle 9	4.98 ± 0.08 c	62.29 ± 6.95 c	784.56 ± 40.50 c
Treatment	CK	6.44 ± 0.13 a	272.47 ± 50.26 a	1716.67 ± 105.01 a
	R	6.46 ± 0.04 a	198.95 ± 12.43 b	1982.26 ± 31.42 a
	B	5.05 ± 0.08 b	70.40 ± 5.60 c	931.03 ± 44.21 b
	G	5.16 ± 0.08 b	65.05 ± 4.80 c	901.16 ± 47.92 b

*CK* rhizosphere soil from control plants without *R. solani* AG8 infection, *R* rhizosphere from plants infected with *R. solani* AG8 in cycle 1, *B* rhizosphere from plants with “bad” treatment (the worst wheat root disease), *G* rhizosphere soil from plants with “good” treatment (the least wheat root disease). The values are means ± standard error. Different letters indicate significant differences for indices (*p* ≤ 0.05, Tukey test). The statistical contrasts were performed separately among cycles and treatments

**Fig. 5:**
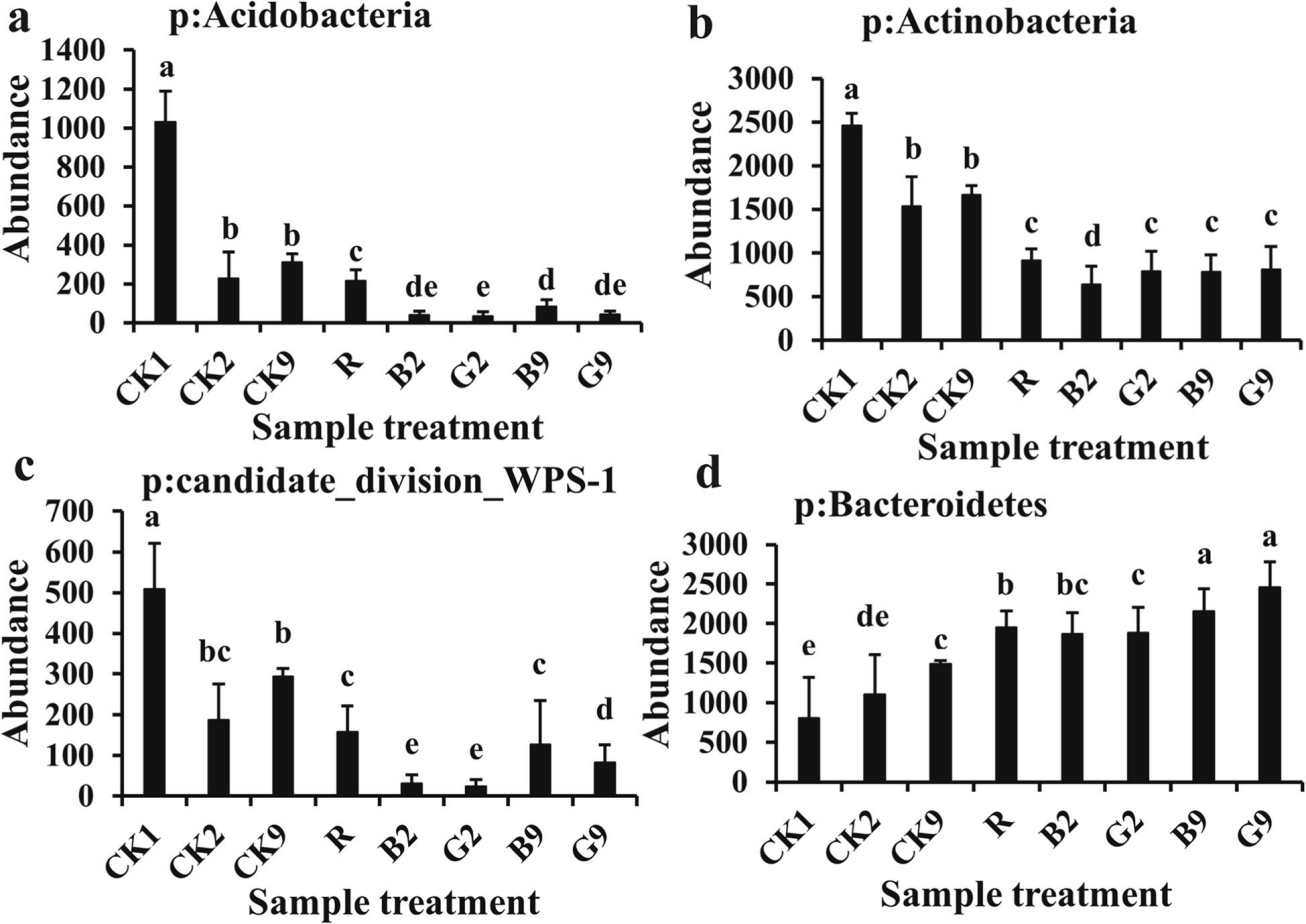
Bacterial phyla influenced by planting cycles and *Rhizoctonia solani* AG8 infection. CK1, CK2, and CK9: rhizosphere soil from control plants without *R. solani* AG8 infection in cycle 1, cycle 2, and cycle 9, respectively; R: rhizosphere soil from plants infected with *R. solani* AG8 in cycle 1; G2, G9: rhizosphere soil from plants with “good” treatment (the least wheat root disease) in cycle 2 and cycle 9, respectively; B2, B9: rhizosphere soil from plants with “bad” treatment (the worst wheat root disease) in cycle 2 and cycle 9, respectively. The values are means ± SE. Different letters indicate significant differences (*p* ≤ 0.05, Tukey’s test)

**Fig. 6:**
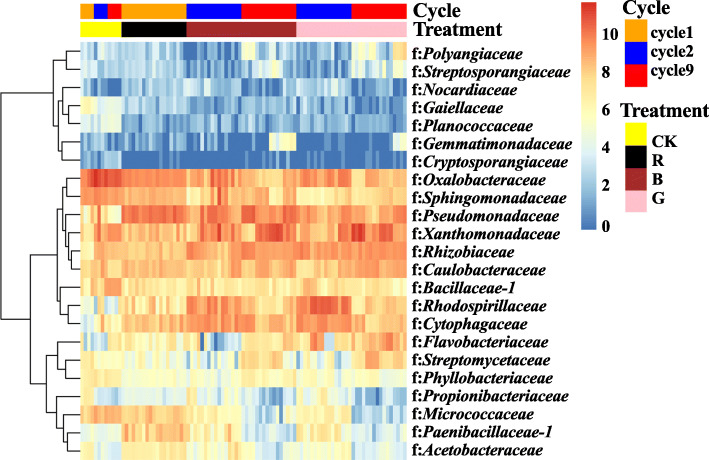
Heatmap of bacterial families significantly influenced by planting cycles and *Rhizoctonia solani* AG8 infection with a *p* value of ≤ 0.05 based on ANOVA of (Log2(1 + *x*)-transformed sequence counts and clustered based on complete-linkage hierarchical clustering of Euclidean distances. Colored bars at the right-top of the graph presented the planting cycles and *R. solani* AG8 inoculation for each sample. CK: rhizosphere soil from control plants without *R. solani* AG8 infection; R: rhizosphere soil from plants infected with *R. solani* AG8 in cycle 1; B: rhizosphere soil from plants with “bad” treatment (the worst wheat root disease); G: rhizosphere soil from plants with “good” treatment (the least wheat root disease)

Phyla Acidobacteria, Actinobacteria, and Candidate division WPS-1 were more abundant in the wheat rhizosphere without *R. solani* AG8 infection than those with AG8 infection after nine planting cycles (Fig. 5a–c), while the phylum *Bacteroidetes* appeared to follow an opposite trend (Fig. 5d) (*p* ≤ 0.05, Tukey’s test). Moreover, the planting cycles and *R. solani* AG8 infection were found to influence soil microbial communities at the family level (Fig. 6). For example, families *Gaiellaceae*, *Planococcaceae*, *Cryptosporangiaceae*, *Oxalobacteraceae*, *Sphingomonadaceae*, *Bacillaceae_1*, *Phyllobacteriaceae*, and *Micrococcaceae* were significantly more abundant from the wheat rhizosphere without AG8 infection (CK) than AG8-infected wheat rhizosphere (R, G, and B) (*p* ≤0.05, Tukey’s test). Among them, the abundance of families *Planococcaceae*, *Cryptosporangiaceae*, and *Bacillaceae_1* in the wheat rhizosphere without AG8 infection (CK) increased over cycles. In contrast, families *Pseudomonadaceae*, *Rhizobiaceae*, *Rhodospirillaceae*, and *Cytophagaceae* were more abundant in the rhizosphere of *R. solani* AG8-infected wheat. In addition, family *Oxalobacteraceae* in AG8-infected wheat dramatically decreased over planting cycles, whereas *Xanthomonadaceae* increased over planting cycles both with and without AG8 infection. Intriguingly, the rhizosphere soils of wheat from “good” (G) treatment were more enriched in *Flavobacteriaceae* than those from “bad” (B) treatment (Fig. 6).

**Fig. 7:**
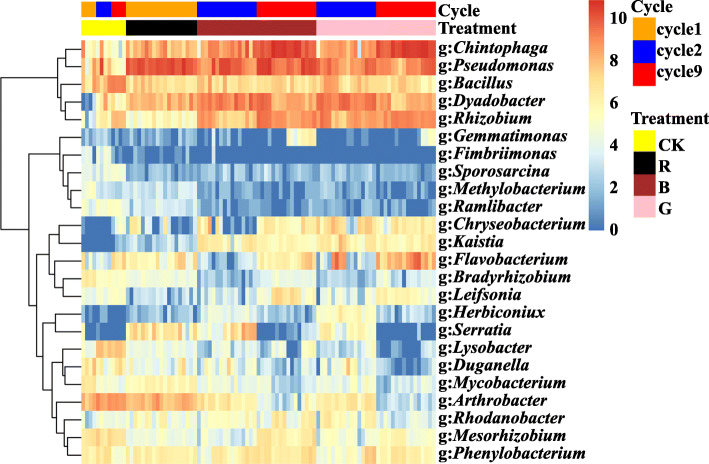
Heatmap of bacterial genera significantly influenced by planting cycles and *Rhizoctonia solani* AG8 infection with a *p* value of ≤ 0.05 based on ANOVA of (Log2(1 + *x*)-transformed sequence counts and clustered based on complete-linkage hierarchical clustering of Euclidean distances. Colored bars at the right-top of the graph presented the planting cycles and *R. solani* AG8 inoculation for each sample. CK: rhizosphere soil from control plants without *R. solani* AG8 infection; R: rhizosphere soil from plants infected with *R. solani* AG8 in cycle 1; B: rhizosphere soil from plants with “bad” treatment (the worst wheat root disease); G: rhizosphere soil from plants with “good” treatment (the least wheat root disease)

After five successive plantings, wheat root damage and shoot stunting were relieved, indicating that disease suppression had developed. Similar results were reported in our previous greenhouse study and a few antagonistic bacteria were successfully isolated from the test samples [43]. Similarly, multi-cycle wheat plantings with *R. solani* AG8 infection recruited some microbial genera which have potential antagonistic activities against phytopathogens in this study (Fig. 7). For example, the genera *Chitinophaga*, *Pseudomonas*, *Chryseobacterium*, *Flavobacterium*, *Serratia*, and *Rhodanobacter* in the rhizosphere of AG8-infected wheat were more abundant than those without AG8 infection, while genera *Bacillus*, *Lysobacter*, *Duganella*, and *Mesorhizobium* exhibited an opposite trend (*p* ≤0.05, Tukey’s test). Moreover, the abundance of *Chitinophaga* from AG8-infected wheat rhizosphere and *Bacillus* from the control samples significantly increased with the planting cycles. In addition, some genera of bacteria which are not known to have antagonistic functions also responded to successive wheat plantings and AG8 infection. Four genera (*Dyadobacter*, *Kaistia*, *Herbiconiux*, and *Phenylobacterium*) were found to be more abundant in AG8-infected wheat rhizosphere, whereas the abundance of six genera (*Fimbriimonas*, *Sporosarcina*, *Methylobacterium*, *Ramlibacter*, *Bradyrhizobium*, and *Arthrobacter*) increased in *R. solani* AG8 non-inoculated soils.

**Fig. 8:**
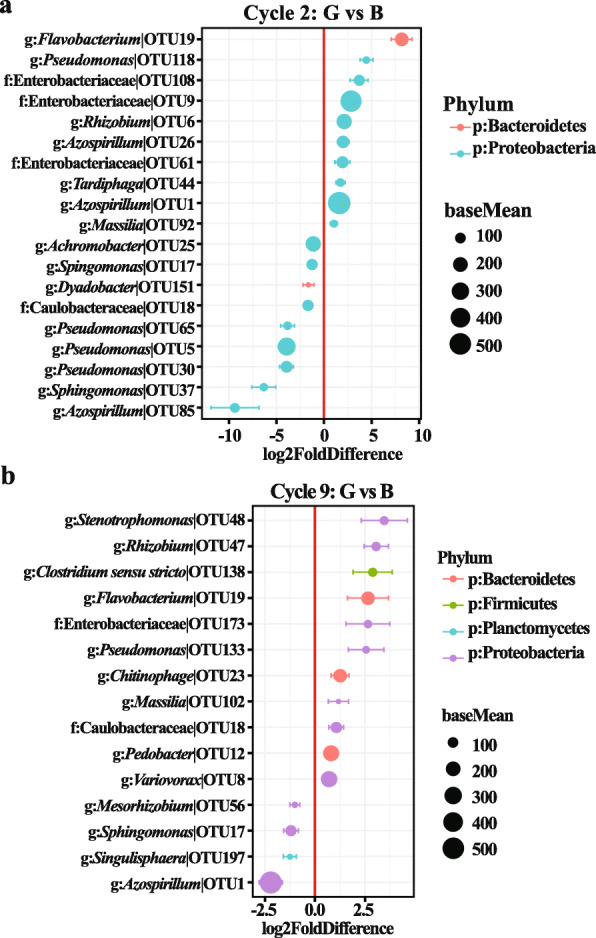
Differentially abundant OTUs identified in the rhizosphere soil from plants between “good” treatment (the least wheat root disease) and “bad” treatment (the worst wheat root disease) in cycle 2 (**a**) and cycle 9 (**b**). Values on the *x*-axis presented the DESeq2-estimated log2-fold difference in the rhizosphere soil from plants between “good” treatment and “bad” treatment samples, where positive values indicate higher abundances in ‘Good ‘ treatment and negative values indicate reduced abundance in “bad” treatment (FDR adjusted *p*-values of < 0.1 were considered to be differentially abundant, Wald’s test). Dots indicate OTUs, where the size of the dot is scaled by its mean abundance among all samples (base mean > 50) and its color represents the phylum to which that OTU belongs. The nearest taxonomy assignment is presented at left. Only OTUs with a mean abundance > 10 and normalized counts > 5 and present in at least 3 samples are presented

DESeq2 analysis identified some OTUs that were differentially abundant between the rhizosphere soils from “good” (G) treatment (the least wheat root disease) and “bad” (B) treatment (the worst wheat root disease) in both cycle 2 and cycle 9 (Fig. 8). Nineteen OTUs from cycle 2 samples and 15 OTUs from cycle 9 samples were influenced by “good” (G) and “bad” treatments, respectively. In cycle 2, 10 OTUs with relative abundances were enriched in wheat rhizospheres from “good” (G) treatment, whereas nine OTUs were abundant in “bad” (B) treatment. In cycle 9, 11 OTUs were enriched in the rhizospheres from the “good” (G) treatment and four were highly abundant in the “bad” (B) treatment. Notably, OTU 19 belonging to the genus *Flavobacterium* consistently increased in rhizospheres from “good” (G) treatment in both cycle 2 and cycle 9, while OTU17 (*Sphingomonas*) was more abundant in “bad” (B) treatment in both cycles. However, OTU1 (*Azospirillum*) and OTU18 (family Caulobacteraceae) showed an opposite pattern between “good” (G) and “bad” (B) treatments in both cycles, and most OTUs varied with planting cycles. Interestingly, the abundance of a group of plant growth-promoting (PGP) microbes, including *Rhizobium*, *Pedobacter*, and *Variovorax*, increased in the rhizosphere from the “good” (G) treatment in cycle 9 (Fig. 8b). Together, these data highlighted that multi-cycle wheat plantings dramatically changed the structure of rhizosphere soil microbial communities, and *R. solani* AG8 application further drove these differences. Furthermore, some plant-beneficial microbial species were enriched with plant growth cycles that may induce suppression of AG8 and enhance plant growth.

### Antifungal capabilities of bacteria in vitro

**Fig. 9:**
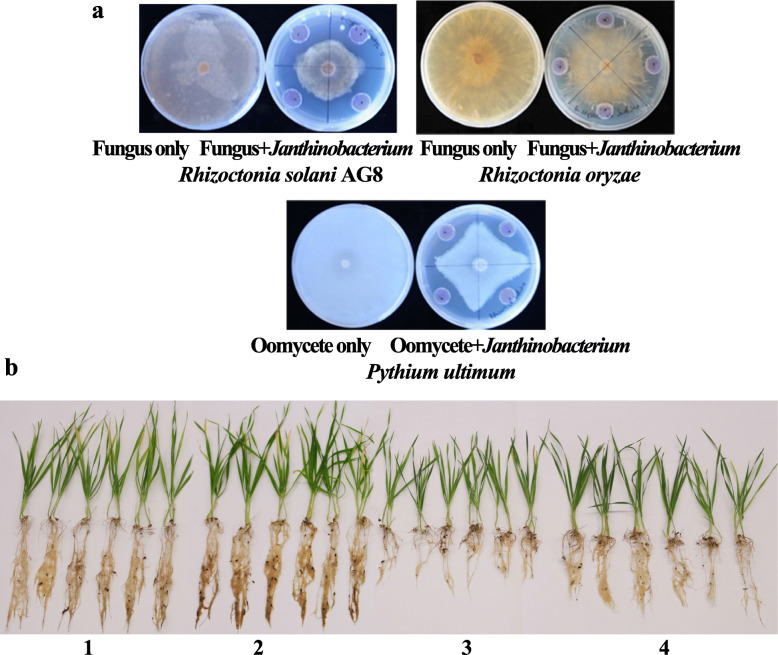
Suppressive activity of *Janthinobacterium* on soilborne pathogens. **a** Dual culture assays *in vitro.* **b** Suppression of *R. solani* AG8 on root rot of wheat plants by *Janthinobacterium*. (1) Wheat grown in Lind soil without *R. solani* AG8 inoculation and wheat seeds untreated with *Janthinobacterium*. (2) Wheat grown in Lind soil without *R. solani* AG8 inoculation and wheat seeds treated with *Janthinobacterium*. (3) Wheat grown in Lind soil with *R. solani* AG8 inoculation. (4) Wheat grown in Lind soil with *R. solani* AG8 inoculation and wheat seeds treated with *Janthinobacterium*. The experiments were conducted three times and showed similar results

To confirm the ability of plants to recruit beneficial bacterial taxa, bacteria were isolated from rhizosphere soils collected from cycles 5, 6, 7, 8, and 9. In this study, a total of 47 bacteria were isolated and categorized into 35 species at a 97% similarity of sequence threshold (Additional file: Table S1). These bacteria were then tested in dual culture assays for their antagonism against soilborne pathogens, *Rhizoctonia solani* AG8, *R. oryzae*, and *Pythium ultimum*. Eleven of 35 bacterial species exhibited antagonistic activities to AG8 at different levels. These were *Pantoea* (OTU951), *Pseudomonas* (OTU163), *Streptomyces* (OTU22), *Chryseobacterium* (OTU993), *Pseudomonas* (OTU118), *Pseudomonas* (OTU245), *Sphingomonas* (OTU2657), *Cupriavidus* (OTU162), *Asticcacaulis* (OTU29), *Rhodococcus* (OTU854) (Fig. S5), and *Janthinobacterium* (OTU131) (Fig. 9a). Six bacterial species, belonging to the genera *Pseudomonas* (OTU163), *Chryseobacterium* (OTU993), *Pseudomonas* (OTU118), *Pseudomonas* (OTU245), *Sphingomonas* (OTU2657) (Fig. S6), and *Janthinobacterium* (OTU131) (Fig. 9a), displayed antagonisms against *R. oryzae* (Fig. 9a). Only one bacterial species, *Janthinobacterium* (OTU131), inhibited the growth of *Pythium ultimum* in ¼ TSA medium (Fig. 9a)*. Janthinobacterium* (OTU131) was used for further study because it exhibited broad antagonistic activities to all three soilborne pathogens. *Janthinobacterium* is a gram-negative bacterium able to produce violacein, a dark purple-violet compound with antimicrobial properties [50] (Fig. S7). When the diluted rhizosphere soil slurries were plated on ¼ TSA medium, more dark-purple colonies were observed from the rhizosphere soil with “good” (G) treatment (the least wheat root disease) than those with “bad” (B) treatment (the worst wheat root disease) (Data not shown). This phenomenon was supported by our sequence data; *Janthinobacterium* (OTU131) were more abundant in wheat rhizospheres from “good” (G) treatment (relative abundance: 1.84 ± 0.01%, mean ± SE) than those from “bad” (B) treatment (relative abundance: 0.52 ± 0.01%) in planting cycle 9. Furthermore, in dual culture assays, the percent inhibition of radial growth (PIRG) values for *R. solani* AG8, *R. oryzae*, and *Pythium ultimum* were 49.63% ± 0.56%, 13.33% ± 1.51%, and 20.12% ± 1.84%, respectively (Table 3).

**Table 3:** Inhibition of radial growth of soilborne pathogens in dual culture by *Janthinobacterium*

Soilborne pathogens	% inhibition of radial growth
*Pythium ultimum*	20.1±1.8
*Rhizoctonia solani* AG8	49.6±0.6
*Rhizoctonia oryzae*	13.3±1.5

The values are means ± standard error of three replicates. The experiments were repeated three times with similar results (Additional file: Table S2)

### Inhibitory effect of *Janthinobacterium* on *Rhizoctonia solani* AG8 in soil

*Janthinobacterium* was further tested to determine its disease suppression activity against *R. solani* AG8 in soil in a greenhouse experiment. The same Lind soil was used, and wheat seeds were treated with the *Janthinobacterium* bacterial slurries (the optical density OD600 value of 1.0) before planting. After 3 weeks growth, compared with the controls (plant seeds untreated/treated with *Janthinobacterium*, but without AG8 infection), three-week-old wheat seedlings were stunted in all *R. solani* AG8-infested soils compared with non-infested, but grew marginally better following the *Janthinobacterium* bacterial treatment. The fresh weight of wheat roots treated with *Janthinobacterium* significantly increased compared with AG8 inoculation only, although it was still significantly less than the controls (Table 4 and Fig. 9b). The shoot fresh weight and length of wheat seedlings were similar in both AG8 inoculation only and AG8 with *Janthinobacterium* treatment, but less or shorter than in the controls.

**Table 4:** The suppression activity of *Janthinobaterium* against *Rhizoctonia solani* AG8 in greenhouse assay

Treatment	Wheat root fresh weight (g)	Wheat shoot fresh weight (g)	Wheat shoot length (cm)
− AG8 − *Janthinobacterium*	251.83 ± 13.54 a	715.43 ± 39.27 a	20.95 ± 0.46 a
− AG8 + seed treated with *Janthinobacterium*	272.92 ± 25.69 a	837.40 ± 29.3 a	20.82 ± 0.45 a
+ AG8 only	145.42 ± 10.79 c	439.92 ± 38.38 b	16.08 ± 0.32 b
+ AG8 + seed treated with *Janthinobacterium*	197.48 ± 12.65 b	596.75 ± 47.48 ab	17.04 ± 0.32 b

“+”: with, “−”: without. The values are means ± standard error of six replicates. The experiments were repeated three times with similar results (Additional file: Table S3). Different letters indicate significant differences (*p* ≤ 0.05, Tukey’s test)

## Discussion

A growing body of research indicates host plants impact root-associated microbial communities [28, 35, 51, 52]. We conducted the multi-cycle plantings with infection by the fungal pathogen *Rhizoctonia solani* AG8 to reveal that successive plantings enhanced disease suppression on wheat and shaped the rhizosphere bacterial communities. Application of the pathogen AG8 further drove the differences of the wheat-associated bacterial communities. Moreover, the bacterial communities in the wheat rhizospheres from “good” (G) treatment (the least wheat root disease) gradually separated from those from “bad” (B) treatment (the worst wheat root disease) over the planting cycles. Most notably, some bacterial species were isolated from the wheat rhizospheres over multi-cycle growth and displayed antagonistic activities to soilborne fungal pathogens. Among them, a species of *Janthinobacterium* exhibited broad-spectrum antagonism against *R. solani* AG8, *R. oryzae*, and *Pythium ultimum* in a dual culture assay and against AG8 in soil. Overall, these findings suggest that repeated monocultures and AG8 infestation could change the root microbiome structure and recruit beneficial microbiota which promote plant growth and reduce soilborne pathogens, and eventually might induce disease-suppressive soils.

Multi-cycle wheat plantings accompanied by rhizosphere microbiota transfers reduced root rot disease caused by *R. solani* AG8 and the root disease suppression was enhanced over successive growth cycles. Soils suppressive to *Rhizoctonia* were similarly reported in our previous study [43] and in the agricultural fields in Australia and the Pacific Northwest in the USA [53–55]. Further studies of disease suppressive soil suggested that suppression resulted from the shifts in microbial community composition and activity, enhancing several groups of bacteria including *Pantoea agglomerans*, *Exiguobacterium acetylicum*, and *Microbacteria* [56]; *Asaia* spp. and *Paenibacillus borealis* [57]; and *Flavobacterium*, *Chryseobacterium*, and *Chitinophaga* [43]. In this study, some bacterial genera that have potential disease suppressive activities were significantly affected by the plant growth cycles. For example, bacteria within the genus *Bacillus* significantly increased with plant growth cycles without AG8 infection. It is well documented that *Bacillus* spp. secrete several metabolites not only to trigger plant growth but to inhibit pathogenic microbial growth in soil or kill pathogens through degrading the cell walls [58–60]. The abundance of *Chitinophaga* was significantly higher in AG8-infested wheat rhizosphere in cycle 9 than those in cycle 2 and cycle 1 (Fig. 7), which is consistent with our previous study [43]. In addition, bacterial species diversity and richness were observed to significantly decline with increasing growth cycle. This might be partially due to starting with cycle 2, in which pasteurized soil was used, which kills or removes some harmful microorganisms compared with the native Lind soil in control and cycle 1. However, the same pasteurization treatment was used for the second cycle and following cycles, and the reduction of bacterial species diversity was still observed over the growth cycles. This could be due to the shifts of bacterial communities driven by the plant and AG8 to favor certain bacterial species over others, leading to reduced bacterial diversity and more plant-specific communities.

Disease suppressive soils typically develop after a disease outbreak [55, 61–64]. This phenomenon is often attributed to plants changing the structure of the microbial community and recruiting protective microbiota in the rhizosphere in response to pathogen attack by producing chemical compounds. In our multi-cycle wheat planting selection system, the soilborne fungal pathogen AG8 was inoculated into soil. The bacterial communities recruited to the AG8-infected rhizosphere were distinct from those without AG8 infection. AG8 infection also enhanced bacterial community separation during cycling indicating the pathogen application modified changes in microbial community composition driven by successive plantings. Similar changes were reported in other studies [64]. For instance, tomato plants challenged with the pathogen *Ralstonia solanacearum* revealed that the soil microbial abundances were changed through the plant root exudation in infected plants [65]. Barley plants challenged with *Fusarium graminearum* enriched the rhizosphere microbiome with potentially antifungal microbes [41]. There is increasing evidence that plants produce compounds to attract beneficial microbes or stimulate the expression of antifungal genes to react to pathogen infection. Therefore, plants can acclimate to biotic stress [39, 41, 66].

Interestingly, *R. solani* AG8 infection increased the abundance of some genera that have suppressive or antagonistic functions, such as *Chitinophaga*, *Pseudomonas*, *Chryseobacterium*, *Flavobacterium*, *Serratia*, and *Rhodanobacter*. Similar results were reported in our previous study [43]: *Chitinophaga*, *Flavobacterium*, and *Chryseobacterium* were more abundant in the rhizosphere of diseased plants infected by *R. solani* AG8 than those of healthy plants. Moreover, some strains of *Flavobacterium* and *Chryseobacterium* produce antimicrobial compounds and stimulate plant immune systems and have been used as bioremediation agents [67]. Most recently, Nishioka et al. [68] recovered *Flavobacterium* species from the rhizosphere soils of the *Allium* plants that suppressed Fusarium wilt on cucumber seedlings and demonstrated that the *Flavobacterium* isolates inhibited the multiplication of the pathogen in soil. *Flavobacterium* was also found to be one of the most abundant bacterial genera present in the soil of banana fields in which Fusarium wilt decline had occurred [69]. The genus *Serratia* belongs to the family *Enterobacteriaceae* within the *Gammaproteobacteria*. *Serratia plymuthica* is a ubiquitous gram-negative bacterium, most frequently associated with plants and used as a broad-spectrum biocontrol agent because it produces antimicrobial compounds [70–72] and was successfully developed as a commercial product called Rhizostar (produced by E-nema GmbH Raisdorf, Germany). Recently *Serratia marcescens* was found to produce several hydrolytic enzymes and showed antagonistic activity against eight fungal pathogens of tea [73]. In contrast, genera *Bacillus*, *Duganella*, and *Lysobacter* were more highly abundant in the rhizosphere soil without AG8 infection. Some strains of *Bacillus* can suppress pathogen-derived microbe-associated molecular patterns (MAMPs)-triggered root immune responses and protect *Arabidopsis* against pathogens [74]. In a previous field study [43] *Duganella* was more abundant in diseased plant rhizospheres, indicating *Duganella* may have different behavior in specific conditions*. Lysobacter* is a chitinolytic bacterium and has potential antagonistic activity against *Rhizoctonia* and nematodes [75–77]. Collectively, the data indicate that upon pathogen attack, pathogen-stressed plants may undergo changes in metabolic pathways and modulate the chemical composition of their rhizospheres, which recruit beneficial and antagonistic bacterial communities. The accumulation of antagonistic microbes can protect plants against the pathogens that initiated the recruitment. In addition, the abundance of other genera was also changed by AG8 infection in this study. Most of them are non-antagonistic bacteria or their biological functions are still unknown. Interestingly, Fujiwara et al. [78] reported that a community of seven non-antagonistic bacterial strains, including one *Kaistia* strain, suppressed the fungal phytopathogen *Fusarium oxysporum* and morphological observations showed the formation of swollen *F. oxysporum* cells in the presence of these bacterial pairs. It demonstrated that complex interactions among apparently non-antagonistic bacteria can result in antagonism against pathogens. Thus, these uncharacterized emergent functions of bacterial consortia may also contribute to suppression activities but require further investigation. Taken together, our results provide further evidence that under the pressure of pathogen attack, plants can enrich beneficial microorganisms to suppress pathogens in the rhizosphere.

In our multi-cycle selection system, wheat seedlings with roots showing relatively more or less disease to *R. solani* AG8 were screened from each cycle and used as rhizosphere inoculants for the following cycle, thus forming the two groups, “good” (G) (the least wheat root disease) treatment and “bad” (B) (the worst wheat root disease) treatment. In the first two cycles, wheat displayed severe stunting. Starting from cycle 3, suppressiveness to AG8 gradually developed and was notable by cycle 5, but the disease severity was still variable among four replicates from “good” or “bad” treatments. To achieve uniform disease symptoms among replicates, the planting was continued and by the 9th cycle all wheat plants of two treatments were tolerant to AG8 infection, but the “good” treatment showed slightly less disease symptoms than the “bad” treatment. Consistent with the disease phenotype, sequence analysis found that the microbial communities separated gradually over the growth cycles. The different quantities of diseased roots likely affect the microbial community composition, but other factors may also contribute. Although care was taken to water the plant containers equally, those with more severe root disease had reduced root systems and typically retained more moisture towards the end of the cycles. Thus, water content may influence soil microbiomes and the development of suppressive soils, something which should be addressed further. Furthermore, DESeq2 analysis found that some OTUs were differentially abundant in the rhizosphere soil between “good” (G) and “bad” (B) treatment in both cycle 2 and cycle 9. Interestingly, the “good” treatment in cycles 2 and 9 was highly enriched the antagonistic microbe OTU 19 belonged to genera *Flavobacterium* [68]. In addition, a group of plant growth-promoting (PGP) microbes, including *Rhizobium*, *Pedobacter*, and *Variovorax*, were highly abundant in the rhizosphere soil from “good” (G) treatment in cycle 9. PGP microbes have shown potential to promote plant growth at different stages via a wide variety of mechanisms [79]. For example, *Rhizobium* is a gram-negative soil bacterium and promotes plant growth through establishing nitrogen-fixing symbiosis with leguminous plants and increasing soil fertility [80]. Another PGP microbe, *Pedobacter* is also capable of colonizing roots of many crops, such as oilseed rape, potato, and strawberry, and could be used as a biofertilizer [81–83]. *Variovorax* is a metabolically diverse genus of plant growth-promoting rhizobacteria which belongs to the family *Comamonadaceae*. *Variovorax* sp. promoted plant growth via producing plant growth substances and enzymes such as siderophores and ACC deaminase [84]. These results suggest that both antagonistic and PGP microbes might contribute to the improvement of wheat growth and tolerance to AG8. It is widely accepted that root exudates play a crucial role in the establishment of the root microbiome [85, 86] and different root exudates are thought to secrete chemical compounds to select specific microbial populations. In our study, although the same wheat cultivar was used, continuous screening of wheat with more tolerance or susceptibility to AG8 might change the components or amounts of chemical compounds or root signals which attract favorite microorganisms [33, 87]. Further efforts to analyze the root exudate composition of each cycle may greatly improve our understanding of the role of plants on the changes of microbial communities and elucidate the mechanisms underlying the recruitment of antagonistic bacteria by plant, and eventually lead to the development of eco-friendly soilborne pathogens management strategies.

*In vitro* bacterial isolation and antifungal capability testing found that eleven of 35 bacterial species inhibited the growth of *R. solani* AG8. Six of them suppressed *R. oryzae* and only one for *Pythium ultimum*. Testing further indicated that AG8 infection is a major driver for the colonization of those antagonistic isolates. Most of them, such as *Pseudomonas*, *Streptomyces*, and *Chryseobacterium*, have been well-documented as pathogen suppressive [43, 52, 61]. Interestingly, a *Janthinobacterium* produces a dark-purple compound, violacein [88]. More dark-purple colonies were observed in the rhizosphere from “good” (G) treatment (the least disease rhizosphere soil) than those from “bad” (B) treatment (the worst disease rhizosphere soil), indicating *Janthinobacterium* was more highly associated with rhizospheres of plants more tolerant to AG8. *Janthinobacterium* is a gram-negative aerobic bacterium, which belongs to the family Oxalobacteraceae of the Class Betaproteobacteria, and commonly exists in soil and aquatic habitats. As a secondary metabolite, violacein has been reported to have antifungal effects [89, 90]. However, Haack et al. [91] revealed that violacein was not the primary cause of the fungal growth inhibition by expressing violacein encoded gene vioABCDE in *E. coli* which had no significant inhibition on *Fusarium graminearum* growth and further observed that the fungal growth inhibition was independent of the amount of violacein. Our antifungal capabilities test showed that *Janthinobacterium* has a broad-spectrum antagonism against soilborne pathogens *R. solani* AG8, *R. oryzae*, and *Pythium ultimum*. Further greenhouse assays discovered that *Janthinobacterium* has antagonistic activity against AG8 in soil. Microbial communities have many potential applications in agriculture and medicine, such as pathogen suppression and environmental remediation. With more antagonistic and plant growth-promoting (PGP) microbes being discovered and isolated, synthetic microbial communities might provide plants with stronger disease resistance and growth promotion than single species, thus become more powerful biotechnological tools to improve the sustainability of agro-ecosystems [92]. Our results will provide valuable resources for the development and testing of synthetic microbial consortia in the future.

## Supplementary Information

**Additional file 1.** Methodological Supplement for the V1-V3 hypervariable region of 16S rRNA gene was amplified by PCR.

**Additional file 2: Table S1.** Bacteria isolated from rhizosphere soil in this study.

**Additional file 3: Table S2.** Inhibition of radial growth of soilborne pathogens in dual culture by *Janthinobacterium* (replicates 2 and 3).

**Additional file 4: Table S3.** The suppression activity of *Janthinobaterium* against *Rhizoctonia solani* AG8 in greenhouse assay (replicates 2 and 3).

**Additional file 5: Figure S1.** The phenotype of wheat grown in Lind soil in the growth chamber after the cycle 1. CK: control without *Rhizoctonia solani* AG8 infection.

**Additional file 6: Figure S2.** The phenotype of wheat grown in pasteurized Lind soil in greenhouse after cycle 2. CK: control plants without *Rhizoctonia solani* AG8 infection; G: plants with ‘Good’ treatment (the least wheat root disease); B: plants with ‘Bad’ treatment (the worst wheat root disease).

**Additional file 7: Figure S3.** The phenotype of wheat grown in pasteurized Lind soil in greenhouse after cycle 5. CK: control plants without *Rhizoctonia solani* AG8 infection; G: plants with ‘Good’ treatment (the least wheat root disease); B: plants with ‘Bad’ treatment (the worst wheat root disease).

**Additional file 8: Figure S4.** The phenotype of wheat grown in pasteurized Lind soil in greenhouse after cycle 9. CK: control plants without *Rhizoctonia solani* AG8 infection; G: plants with ‘Good’ treatment (the least wheat root disease); B: plants with ‘Bad’ treatment (the worst wheat root disease).

**Additional file 9: Figure S5.** Dual culture assays *in vitro* for inhibition of growth of *Rhizoctonia solani* AG8 by bacteria isolates on ¼ TSA medium*.*

**Additional file 10: Figure S6.** Dual culture assays *in vitro* for inhibition of growth of *Rhizoctonia oryzae* by bacteria isolates on ¼ TSA medium*.*

**Additional file 11: Figure S7.** Violacein produced by *Janthinobacterium* on TSA medium.

## Data Availability

The raw sequence data was deposited in the SRA of the NCBI (https://www.ncbi.nlm.nih.gov/sra) under accession number PRJNA578725. The datasets during and/or analyzed during the current study available from the corresponding author on reasonable request.
