# Supplementary material for: Rhizosphere community selection reveals bacteria associated with reduced root disease
Source: Microbiome. 2021 Apr 9;9:86. doi: 10.1186/s40168-020-00997-5 (PMC8035742; doi:10.1186/s40168-020-00997-5)
Supplement: Supplementary file 2 — Additional file 1. Methodological Supplement for the V1-V3 hypervariable region of 16S rRNA gene was amplified by PCR. [file 40168_2020_997_MOESM2_ESM.docx]

The V1-V3 hypervariable region of the 16S rRNA gene was amplified with primers MN_27F (5’-AGAGTTTGATCMTGGCTCAG-3’) and MN_534R (5’-ATTACCGCGGC TGCTGG-3’) using a dual-indexing approach. Briefly, the first round of PCR was performed as follows: an initial denaturing at 95°C for 5 min, followed by 25 cycles of 98°C for 20 s, 55°C for 15 s, and 72°C for 1 min, with a final extension at 72°C for 1 min. The products from the first PCR were diluted 1:100, and 5 μl was included in a second round of PCR using indexing primers. The second round of PCR was performed as follows: an initial denaturation at 95°C for 5 min, 10 cycles of 98°C for 20 s, 55°C for 15 s, and 72°C for 1 min, with a final extension at 72°C for 5 min.
