# Supplementary figures and images for: Rhizosphere community selection reveals bacteria associated with reduced root disease
Source: Microbiome. 2021 Apr 9;9:86. doi: 10.1186/s40168-020-00997-5 (PMC8035742; doi:10.1186/s40168-020-00997-5)

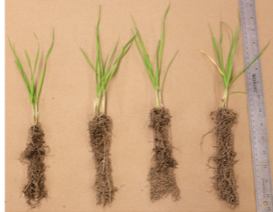

**CK (-AG8)**

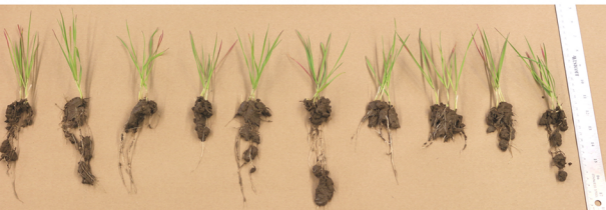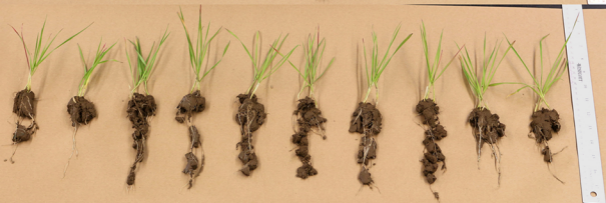

**Treatment (+AG8)**

**Cycle 1**

Supplement: Supplementary file 6 — Additional file 5: Figure S1. The phenotype of wheat grown in Lind soil in the growth chamber after the cycle 1. CK: control without Rhizoctonia solani AG8 infection. [file 40168_2020_997_MOESM6_ESM.pdf]

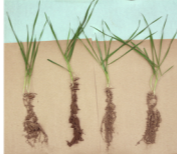

**CK (-AG8)**

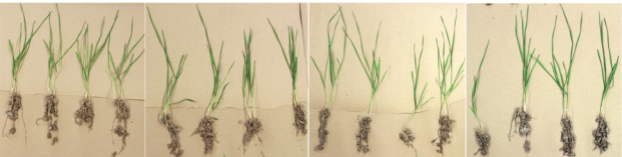

**G (+AG8)**

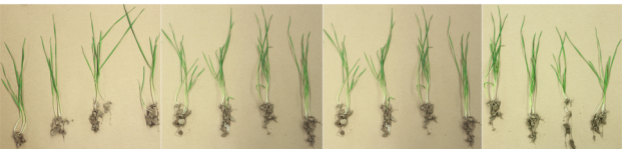

**B (+AG8)**

**Cycle 2**

Supplement: Supplementary file 7 — Additional file 6: Figure S2. The phenotype of wheat grown in pasteurized Lind soil in greenhouse after cycle 2. CK: control plants without Rhizoctonia solani AG8 infection; G: plants with ‘Good’ treatment (the least wheat root disease); B: plants with ‘Bad’ treatment (the worst wheat root disease). [file 40168_2020_997_MOESM7_ESM.pdf]

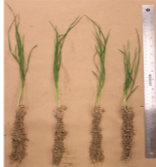

**CK (-AG8)**

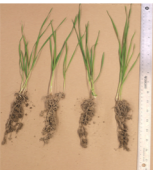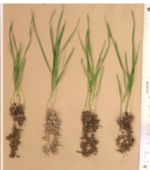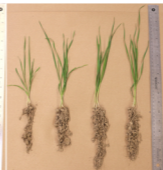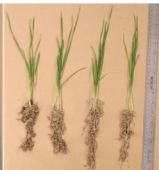

**G (+AG8)**

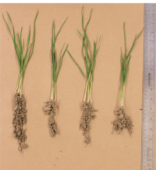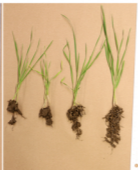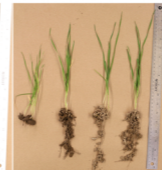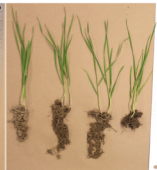

**B (+AG8)**

**Cycle 5**

Supplement: Supplementary file 8 — Additional file 7: Figure S3. The phenotype of wheat grown in pasteurized Lind soil in greenhouse after cycle 5. CK: control plants without Rhizoctonia solani AG8 infection; G: plants with ‘Good’ treatment (the least wheat root disease); B: plants with ‘Bad’ treatment (the worst wheat root disease). [file 40168_2020_997_MOESM8_ESM.pdf]

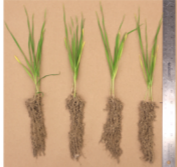

**CK (-AG8)**

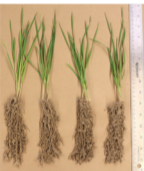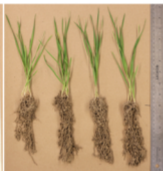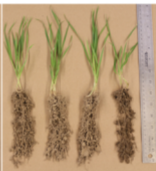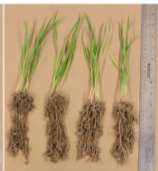

**G (+AG8)**

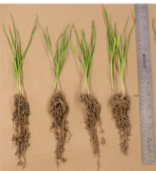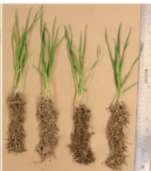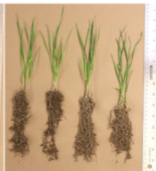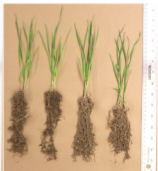

**B (+AG8)**

**Cycle 9**

Supplement: Supplementary file 9 — Additional file 8: Figure S4. The phenotype of wheat grown in pasteurized Lind soil in greenhouse after cycle 9. CK: control plants without Rhizoctonia solani AG8 infection; G: plants with ‘Good’ treatment (the least wheat root disease); B: plants with ‘Bad’ treatment (the worst wheat root disease). [file 40168_2020_997_MOESM9_ESM.pdf]

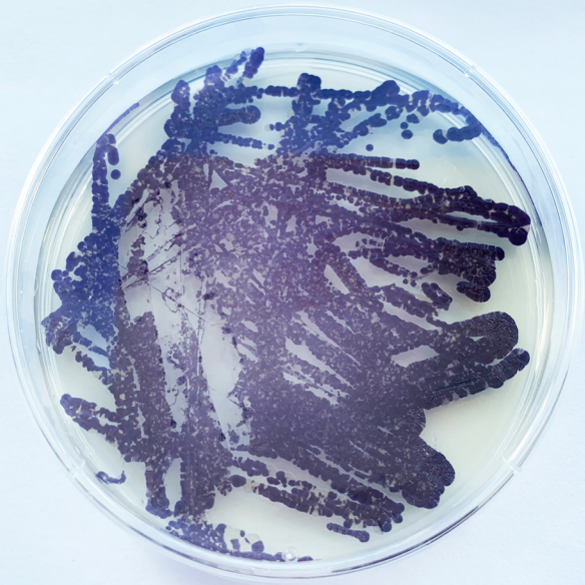

Supplement: Supplementary file 12 — Additional file 11: Figure S7. Violacein produced by Janthinobacterium on TSA medium. [file 40168_2020_997_MOESM12_ESM.pdf]
