# Supplementary material for: Rhizosphere community selection reveals bacteria associated with reduced root disease
Source: Microbiome. 2021 Apr 9;9:86. doi: 10.1186/s40168-020-00997-5 (PMC8035742; doi:10.1186/s40168-020-00997-5)
Supplement: Supplementary file 10 — Additional file 9: Figure S5. Dual culture assays in vitro for inhibition of growth of Rhizoctonia solani AG8 by bacteria isolates on ¼ TSA medium. [file 40168_2020_997_MOESM10_ESM.pdf]

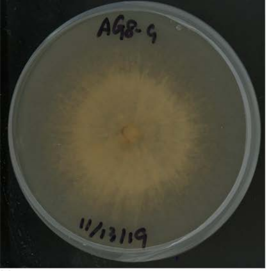

***Rhizoctonia solani* AG8**

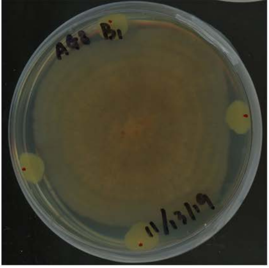

***Pantoea* (OTU951)**

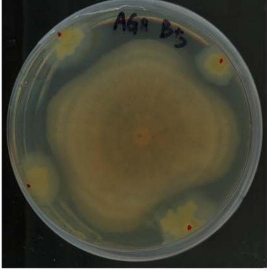

***Pseudomonas* (OTU163)**

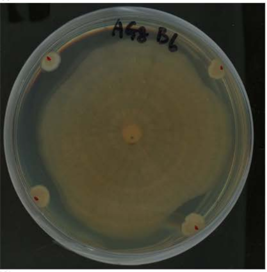

***Streptomyces* (OTU22)**

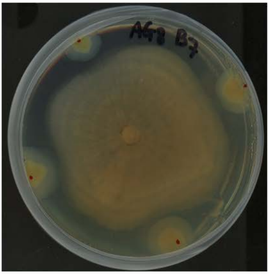

***Chryseobacterium* (OTU993)**

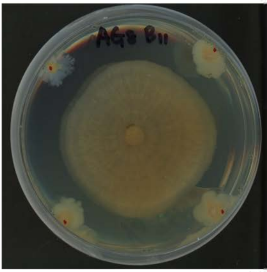

***Pseudomonas* (OTU118)**

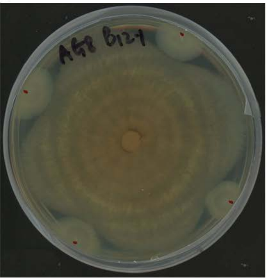

***Pseudomonas* (OTU245)**

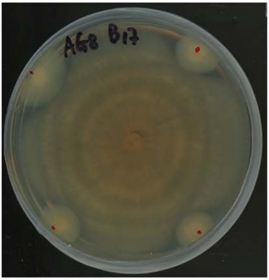

***Sphingomonas* (OTU2657)**

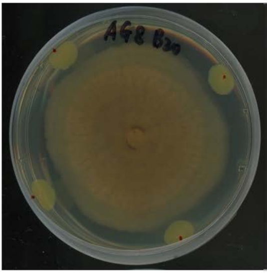

***Cupriavidus* (OTU162)**

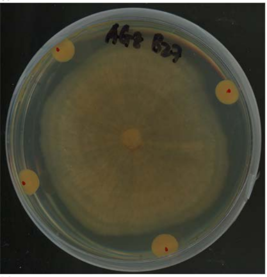

***Asticcacaulis* (OTU29)**

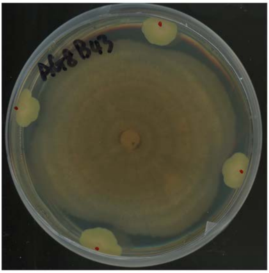

***Rhodococcus* (OTU854)**
