# Supplementary material for: Rhizosphere community selection reveals bacteria associated with reduced root disease
Source: Microbiome. 2021 Apr 9;9:86. doi: 10.1186/s40168-020-00997-5 (PMC8035742; doi:10.1186/s40168-020-00997-5)
Supplement: Supplementary file 11 — Additional file 10: Figure S6. Dual culture assays in vitro for inhibition of growth of Rhizoctonia oryzae by bacteria isolates on ¼ TSA medium. [file 40168_2020_997_MOESM11_ESM.pdf]

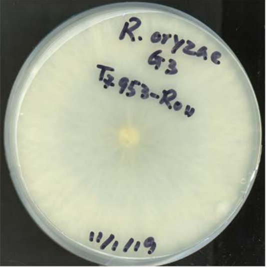

Rhizoctonia oryzae

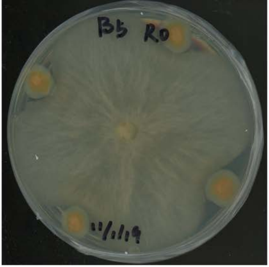

Pseudomonas (OTU163)

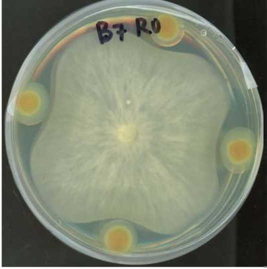

Chryseobacterium (OTU993)

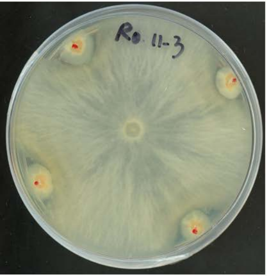

Pseudomonas (OTU118)

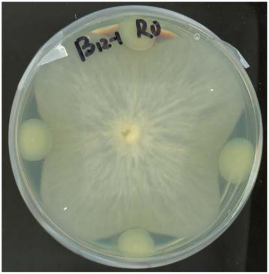

Pseudomonas (OTU245)

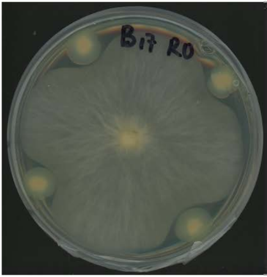

Sphingomonas (OTU2657)
